# A Clinical, Pharmacological, and Formulation Evaluation of Melatonin in the Treatment of Ocular Disorders—A Systematic Review

**DOI:** 10.3390/ijms25073999

**Published:** 2024-04-03

**Authors:** Alessia Romeo, Adrienn Kazsoki, Teresa Musumeci, Romána Zelkó

**Affiliations:** 1 Department of Drug and Health Sciences, University of Catania, Via Santa Sofia 64, 95125 Catania, Italy; alessia.romeo@phd.unict.it (A.R.); tmusumec@unict.it (T.M.); 2 University Pharmacy Department of Pharmacy Administration, Semmelweis University, Hőgyes Endre Street 7–9, 1092 Budapest, Hungary; kazsoki.adrienn@semmelweis.hu

**Keywords:** melatonin, ocular drug delivery system, ophthalmic insert, clinical evaluation, pharmacology, systematic analysis, PRISMA 2020

## Abstract

Melatonin’s cytoprotective properties may have therapeutic implications in treating ocular diseases like glaucoma and age-related macular degeneration. Literature data suggest that melatonin could potentially protect ocular tissues by decreasing the production of free radicals and pro-inflammatory mediators. This study aims to summarize the screened articles on melatonin’s clinical, pharmacological, and formulation evaluation in treating ocular disorders. The identification of relevant studies on the topic in focus was performed according to the Preferred Reporting Items for Systematic Reviews and Meta-Analyses (PRISMA 2020) guidelines. The studies were searched in the following databases and web search engines: Pubmed, Scopus, Science Direct, Web of Science, Reaxys, Google Scholar, Google Patents, Espacenet, and Patentscope. The search time interval was 2013–2023, with the following keywords: melatonin AND ocular OR ophthalmic AND formulation OR insert AND disease. Our key conclusion was that using melatonin-loaded nano-delivery systems enabled the improved permeation of the molecule into intraocular tissues and assured controlled release profiles. Although preclinical studies have demonstrated the efficacy of developed formulations, a considerable gap has been observed in the clinical translation of the results. To overcome this failure, revising the preclinical experimental phase might be useful by selecting endpoints close to clinical ones.

## 1. Introduction

Melatonin (MEL) is a neurohormone whose secretion occurs predominantly in the pineal gland located in the brain. The discovery of this molecule dates to the middle of the last century and can be attributed to dermatologist Aaron Lerner and his collaborators, who coined the name melatonin based on its pigmentation effect on the skin (by merging the prefix “mela-” referring to melanin and the suffix “-tonin” denoting the precursor of that molecule, serotonin). Later, the molecule was classified as an indoleamine and identified as N-acetyl-5-methoxytryptamine [1].

Although MEL was initially thought to be exclusively related to the dermatological field, over time it has been shown to be involved in the regulation of a variety of physiological processes. Lerner himself, in the late 1970s, was the first to note that the molecule was able to affect circadian rhythms [2].

Over the years, the hormone has been associated with processes of pubertal development and aging, the modulation of immune response, inflammation, antioxidant defenses, neuroprotection, and the control of blood pressure and temperature [3,4,5,6,7,8,9,10].

MEL exerts its effects by acting on three types of receptors. Two of these are MT1 and MT2 receptors, high-affinity transmembrane G-protein-coupled receptors that share 60% homology. MT1 is distributed in several brain areas (such as the pituitary, hypothalamus, thalamus, cortex, hippocampus, cerebellum, amygdala, substantia nigra, and nucleus accumbens) but has also been identified in ocular structures such as the cornea (epithelium, stroma, endothelium), sclera, and retina (retinal vessels, retinal ganglion cells, and amacrine cells of the inner and outer plexiform layers). MT2 is largely localized in the retina but is also present in the hippocampus, cerebellum, cortex, and paraventricular nucleus [11,12].

The third MT3 receptor has been identified as a quinone reductase 2 enzyme and, unlike the first two, has cytoplasmic localization and low affinity. Although this enzyme is expressed in peripheral structures (heart, liver, kidney, lungs), previous studies have shown its involvement in the modulation of intraocular pressure (IOP) [13,14].

**Figure 1:**
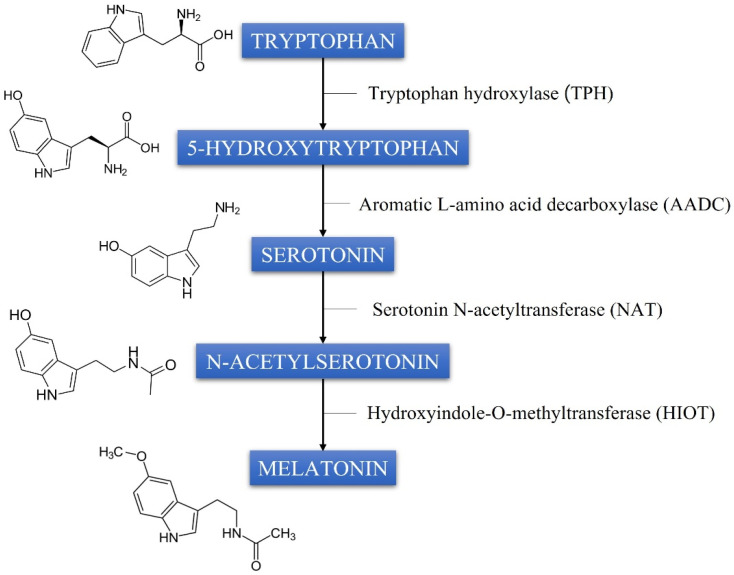
The synthesis of melatonin from the essential amino acid tryptophan.

In addition to the pineal gland, MEL synthesis also occurs in other extrapineal tissues (such as the brain, lens, ciliary body, and retina) and originates from the amino acid precursor tryptophan [15]. The hydroxylation of tryptophan by the enzyme tryptophan hydroxylase (TPH) converts it to 5-hydroxytryptophan. This is then decarboxylated by aromatic L-amino acid decarboxylase (AADC) to produce serotonin. The latter undergoes two further conversions, an acetylation (to N-acetylserotonin) and a methylation involving serotonin N-acetyltransferase (NAT) and hydroxyindole-O-methyltransferase (HIOT), respectively, to produce MEL (Figure 1). Once synthesized, the molecule is released into capillaries and distributed to body tissues [16]. 

Pineal synthesis is closely regulated by the light/dark cycle. During the hours of darkness, norepinephrine released from postganglionic sympathetic fibers reaches the pineal where it activates α- and β-adrenergic receptors, increasing intracellular cAMP levels, NAT enzyme transcription, and thus MEL synthesis. Conversely, during the day, norepinephrine release is attenuated and consequently MEL synthesis too [17].

Although pinealocytes are the cells that synthesize most MEL in the pineal gland, a study showed that the rat retina continues to synthesize MEL even after pinealectomy [11].

It was observed that among organelles and subcellular structures, the highest concentration of MEL was found in mitochondria [18]. Karasek and colleagues observed that the mitochondria volume was greatest during the night and corresponded to the peak of MEL production [19]. These hypotheses, supported by the identification of the enzymes responsible for MEL biosynthesis in the mitochondrial matrix, confirmed that these organelles would be responsible for MEL synthesis [20].

**Figure 2:**
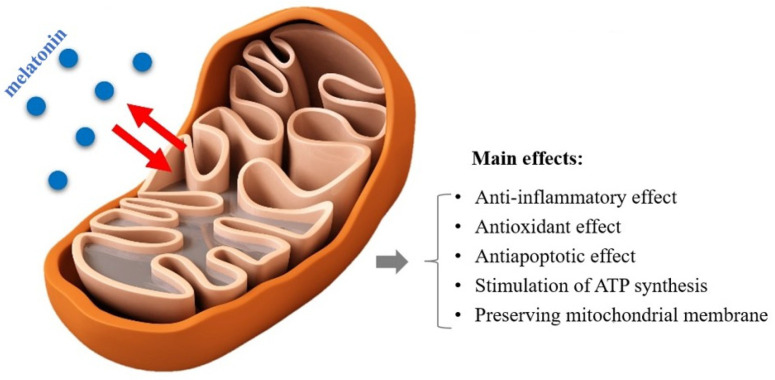
Main effects of melatonin at the mitochondrial level.

Mitochondria play several important roles such as ATP production, the control of cellular metabolism, and the regulation of programmed cell death. The hormone produced at the mitochondrial level exerts numerous beneficial effects with the aim of preserving the function and structure of these organelles (Figure 2). MEL, in addition to stimulating ATP synthesis and preserving membrane potential, counteracts oxidative stress and antiapoptotic events for which these subcellular structures are most responsible [21,22,23].

The eye is a metabolically active structure, and the visual system ranks among the brain systems that in terms of ATP consume the most energy. Although the highest metabolic activity involves the ocular surface (cornea and conjunctiva), which is the most exposed part of the eye, internal tissues (retina and optic nerve) are also subject to dysfunction caused by oxidative stress [24]. Among body tissues, the retina and optic nerve are those that require the greatest energy sustainment, so they are rich in mitochondria. Therefore, mitochondrial dysregulation in these tissues could involve a plethora of pathophysiological conditions and contribute to the pathogenesis of various ocular disorders [25].

In recent decades, the beneficial properties of MEL have attracted much attention in the treatment and prevention of impaired ophthalmic conditions. MEL’s ability to prevent oxidative damage and associated mitochondrial dysfunction has proven to be a useful approach in the management of age-related macular degeneration (AMD), where the molecule has been able to preserve the viability of retinal pigment epithelium (RPE) cells [26,27].

MEL also exerts control over the expression of inflammatory cytokines (IL-6 and TNF-α) and the pathological secretion of vascular endothelial growth factor (VEGF) at the retinal level. The inhibition of the angiogenic factor counteracts the neovascularization that is a manifestation of proliferative diabetic retinopathy; therefore, MEL could be an effective therapeutic resource [28].

Several studies have evaluated the influence of melatonin on the modulation of IOP. It has been found that IOP and MEL follow inverse circadian rhythms; during the day, MEL levels are low while IOP has high values, vice versa during the night. This relationship was confirmed by studies showing that MEL levels were significantly altered in ocular diseases such as glaucoma [29]. Investigations in animal models and clinical studies proved that MEL administration was effective in regulating IOP [30].

In addition, the neuroprotection of injuries caused by ocular hypertension has shown an added efficacy of the therapeutic benefits of this molecule in the management of glaucomatous conditions [31]. Collectively, these mechanisms could be useful in protecting ocular tissues from oxidative damage and subsequent pathologies that might occur.

The importance of summarizing various melatonin formulations lies in optimizing drug delivery to the eye, overcoming bioavailability challenges, and potentially improving therapeutic outcomes for ocular conditions. By exploring different formulations, researchers aim to enhance the efficacy and bioavailability of melatonin for treating eye diseases, which is crucial for developing effective ocular drug delivery systems. While numerous reviews exist regarding the ocular application of MEL, a systematic review has not been put forth thus far. Given this context, we deemed it beneficial to conduct an extensive search across various databases, utilizing a comprehensive approach to gather, assess, and analyze relevant empirical data through meta-analysis, aiming to offer a comprehensive overview of the subject.

## 2. Materials and Methods

The identification of relevant studies on the topic in focus was performed according to the Preferred Reporting Items for Systematic Reviews and Meta-Analyses (PRISMA 2020) guidelines.

### 2.1. Eligibility Criteria

The following criteria were established for the selection of research articles and patents to be included in this systematic review: original research studies published in peer-reviewed journals and published patents were included, while review articles, conference papers, book chapters, and guidelines were excluded. The search was limited to English-language studies published in the time range between 2013 and 2023. This review included all research studies where MEL was administered in the eye. No eligibility criteria were set regarding the diseases treated or regarding the in vitro and in vivo studies conducted. Studies comparing the efficacy of MEL with that of analogs were also included, while articles focused entirely on the delivery of analogous molecules were not considered. 

### 2.2. Search Strategy

The search for articles and patents relevant to the administration of MEL for the treatment of ocular disorders was systematically performed using the same keywords specified below and their equivalent synonyms in the following databases and web search engines: Pubmed, Scopus, Science Direct, Web of Science, Reaxys, Google Scholar, Google Patents, Espacenet, and Patentscope. The search queries were constructed as follows: (melatonin) AND (ocular OR ophthalmic) AND (formulation OR insert) AND (disease). The search results were collected and tabulated as shown in the next chapter. 

### 2.3. Data Collection and Extraction

A PRISMA 2020 flowchart was employed to tabulate and summarize the data collected from the research. Research studies and patents obtained from the consulted databases were exported to the Mendeley reference manager, and duplicates were removed. The remaining articles were subjected to further screening processes by evaluating eligibility based on the title, then the abstract, and finally the full text. Relevant residual articles were consulted to extract the information of interest, which was tabulated under the following items: therapeutic indication, the type of formulation, the route of administration, constituent materials, the concentration of the loaded active, physicochemical characteristics (size, polydispersion index (PDI), zeta potential (ZP), and encapsulation efficiency (EE%)), animal models used in the in vivo studies, and properties of the considered systems. The information extracted from the patents was as follows: the background, target pathology, specific use of MEL, applicant/manufacturer, patent number, and publication date. 

## 3. Results

### 3.1. Studies Included from the Consulted Databases

**Figure 3:**
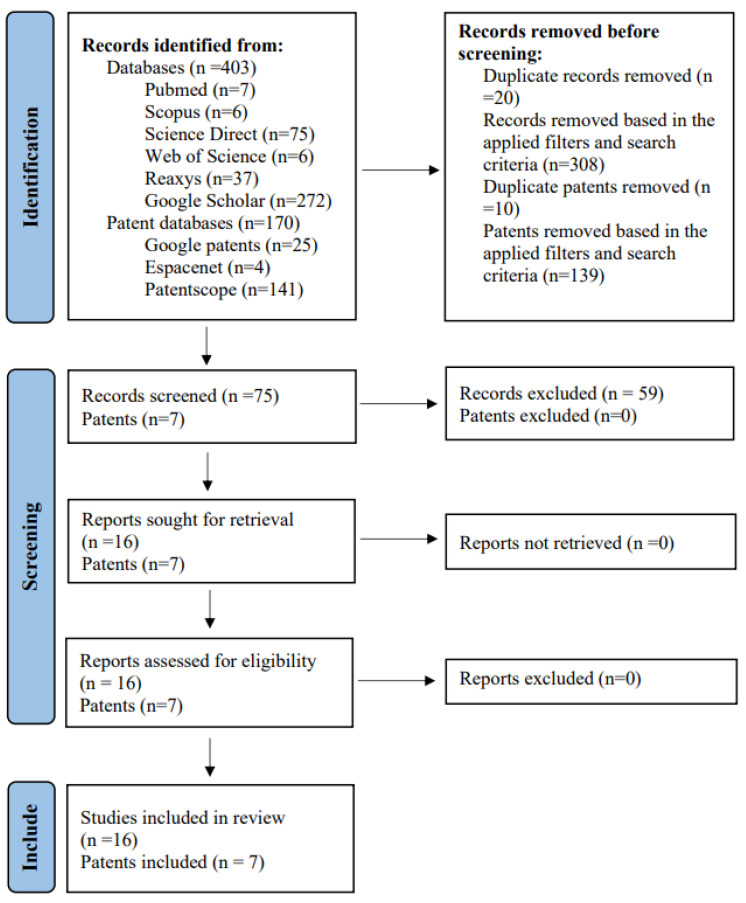
A PRISMA 2020 flow diagram showing relevant articles and patents included in the study.

A total of 403 articles and 170 patents were collected from searches performed on all databases, including 272 from Google Scholar, 75 from Science Direct, 37 from Reaxys, 7 from Pubmed, 6 from Scopus, and 6 from Web of Science. Of the total patents, 141 were obtained from Patentscope, 25 from Google Patents, and 4 from Espacenet. Of the sum total, only 16 articles and 7 patents met the inclusion criteria and thus were included in this review. The flowchart in Figure 3 illustrates the steps of identification, screening, and inclusion. 

### 3.2. The Results of the Studies

#### 3.2.1. Melatonin Ophthalmic Indications and Drug Delivery Strategies

**Figure 4:**
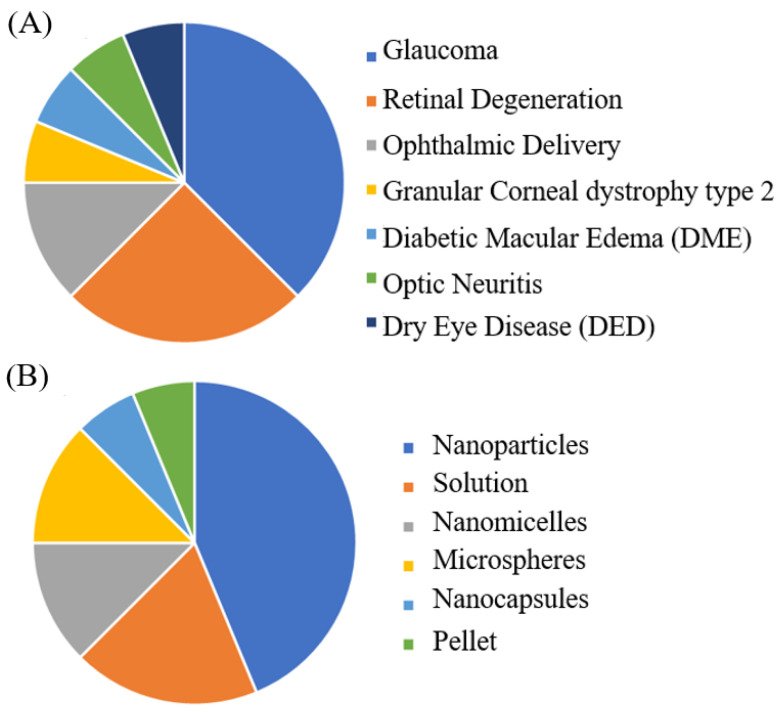
A PRISMA chart analysis of the therapeutic indications (**A**) and types of systems (**B**) explored in the consulted articles.

Based on the above criteria, the articles considered relevant were reviewed and are listed in Table 1. The included studies were classified according to the formulation type and therapeutic indication; a summary of the gathered information is depicted in Figure 4. From Figure 4A, it is evident that the largest number of studies for the ocular delivery of MEL had designed formulations intended for the treatment of conditions such as glaucoma and retinal degeneration. A smaller percentage also developed formulations for the treatment of ocular disorders such as granular corneal dystrophy type 2 (GCD2), diabetic macular edema (DME), optic neuritis, and dry eye disease (DED). In contrast, in Figure 4B, it was found that the most investigated systems for MEL ocular delivery consisted of nanoparticles (NPs).

According to the information from the consulted studies, it was observed that in vivo experiments on laboratory animals involved mice (14.3%), rats (28.6%), and albino rabbits (57.1%). The primary objective of the studies was to increase corneal permeation and absorption to improve the bioavailability of MEL. The results showed the possibility to modulate MEL release from formulations compared to solutions, permitting sustained drug release. In addition, increased permeation was reported for the reviewed systems, whereby the formulations showed good ocular tolerability in vivo.

#### 3.2.2. Ophthalmic Conditions Treated with Melatonin

A summary of published studies over the past 10 years on the use of MEL in the ocular field is discussed below. Given the important role that MEL has shown in the control of intraocular pressure, several studies have investigated the use of this molecule for the treatment of glaucomatous conditions. 

The MEL neuroprotected effect was also tested on a model of DME, which is one of the primary causes of blindness in diabetic retinopathy. The study in question did not involve the incorporation of MEL into any vector but aimed to investigate the protective mechanisms that MEL exerted on an in vitro model of DME using human retinal pigmented epithelium cells. The results showed that MEL supported the integrity of the blood–retinal barrier by suppressing angiogenesis and apoptosis, while at the mitochondrial level, MEL preserved homeostasis by regulating the expression of genes involved in fission, mitophagy, and mitochondrial biogenesis [45].

A MEL-loaded plant oil-based pellet intended for subcutaneous implantation was used to evaluate the activity of the molecule on an inflammatory condition of the optic nerve, optic neuritis. This condition involves decreases in the pupillary light reflex and alterations in circadian physiology. MEL was able to both counteract the decrease in the pupillary light reflex and restore the altered circadian rhythm [46].

In addition to diseases affecting the posterior eye segment, MEL has also been applicable in treating ocular surface disorders such as DED, a multifactorial condition that, even when treated, often does not provide rapid relief [48]. In order to restore tear film homeostasis and provide protection to ocular epithelial cells, Jin et al. proposed the co-delivery of MEL and tavilermide, a small molecule able to preserve tear film integrity, in mesoporous polydopamine NPs. The synergistic treatment strategy with the multifunctional eye drops promoted mucin secretion and supported the restoration of ocular surface cells, speeding up the alleviation of DED [47]. 

MEL has been shown to potentially offer therapeutic benefits for ocular surface condition GCD2. It exhibits a dual antioxidant effect by safeguarding corneal fibroblasts through direct ROS scavenging and by modulating antioxidant enzymes, as demonstrated in studies [49].

#### 3.2.3. Melatonin Formulations

Table 1 summarizes the materials selected to produce delivery carriers improving adsorption, stability, and provided protection against the chemical and physical degradation of the molecule. Polymeric (PLGA, PLA, PLGA-PEG, Soluplus^®^, and polydopamine), lipid (Softisan^®^, DDAB, CTAB, TCT), protein (HSA), oligosaccharide (HPβCD), and vegetable (vegetable oils) materials were used in the reviewed formulations. All these materials were biodegradable and biocompatible, ideal requirements for achieving excellent in vivo tolerability. Overall, the delivery systems examined provided enhanced drug permeation into target tissues and the sustained release of the molecule, factors that contributed to the improved delivery and therapeutic efficacy of MEL.

To circumvent the limitations of poor bioavailability associated with conventional aqueous eye drops, poly(d,l-lactide-co-glycolide) (PLGA) and PLGA-poly(ethylene glycol) (PLGA-PEG) NPs were prepared by a solvent displacement method and were studied in vivo to assess ocular tolerability and measure IOP. The two polymers used were selected, in addition to their proven biocompatibility and biodegradability properties, to ensure the sustained release of MEL and particularly the PLGA-PEG copolymer to increase mucoadhesiveness with the ocular surface. In all formulations, both in liquid and freeze-dried dosage forms, MEL was successfully encapsulated. Although all NPs showed suitable technological characteristics for ophthalmic application, pegylated NPs showed superior performance in the treatment of glaucoma. The PLGA-PEG NPs, compared with the aqueous solution of MEL and PLGA NPs, provided more effective (a maximum reduction of 5 mmHg) and prolonged (persistent up to 8 h) IOP lowering, confirming the hypothesis that the mucoadhesive properties of the copolymer could have increased the contact time with the ocular mucosa [32].

NPs of PLGA for glaucoma treatment were also successfully fabricated for the co-delivery of MEL and dexamethasone (DX). These systems were prepared and simultaneously loaded with both molecules using the coaxial electrospray technique. The use of this technique resulted in a high loading capacity for both drugs in systems with core-shell morphology, where DX was encapsulated in the shell and MEL in the core. In vitro studies showed sustained and nontoxic release on retinal cells for both drugs, while in vivo studies showed that molecules loaded in NPs exhibited better corneal penetration compared to free molecules. The use of the two molecules in combination was able to provide effective antioxidant and hypertensive effects, which were useful in preserving the loss of retinal ganglion cells (RGCs) and hypertension phenomena [36].

Another type of NP investigated for MEL delivery in glaucoma treatment was cationic solid lipid NPs (SLNs). These lipid nanosystems were produced by a quasi-emulsion solvent diffusion (QESD) method, which was generally used for the preparation of polymeric NPs. The lipid matrix consisted of Softisan^®^ 100 mixed with lipid modifiers (palmitic acid and stearic acid) to improve the fluidity. With the aim of increasing the mucoadhesiveness of SLNs, the surface was decorated with the cationic lipid didodecyldimethylammonium bromide (DDAB). These nanosystems showed good ocular tolerability in addition to enhancing the pharmacological effect of MEL. It was reported that a single topical application was able to effectively reduce (a maximum reduction of 7 mmHg) IOP for up to 24 h [33].

Further evidence that nanomicellar formulations of MEL were effective in the treatment of glaucoma was reported in two studies conducted by Dal Monte et al [37]. In the first study, the hypotonizing effect of MEL formulated in nanomicelles or in saline solution was examined. MEL-loaded nanomicellular formulations were compared with nanomicelles loaded with the agomelatine analog, either alone or in combination with MEL. The addition of lipoic acid, a natural antioxidant, was also evaluated. Although the molecule alone did not show hypertensive effects on glaucoma murine models, the study aimed to evaluate the influence on the hypotonizing effect of melatoninergic compounds [50]. The results showed that the combination of MEL and agomelatine potentiated the magnitude of the hypotonizing effect compared with each compound alone and that the addition of lipoic acid ensured a longer duration of the lowering effect [35]. The second study aimed to evaluate the neuroprotective effect of the MEL/agomelatine nanomicellular formulation on a rat model of hypertensive glaucoma. Although the formulation was effective in counteracting the loss of RGCs and promoting functional recovery, it was not fully clarified whether the neuroprotective activity was direct or secondary to the hypotensive effect [37].

The formulations discussed so far were intended for topical administration and had nanometer sizes smaller than 500 nm. Regarding the intraocular administration of drugs, delivery systems with different sizes, selected according to the target site, pathology, and treatment period, could be used. The intravitreal injection of microsystems (1–1000 μm) in suspension could be useful to sustain prolonged therapeutic concentrations [51]. Among them, microspheres (MSs) have gained increasing interest for the treatment of intraocular disorders. However, it has been reported that the intravitreal administration of amounts greater than 0.1 mg of PLGA MS can induce retinal dysfunction in rodents [52].

To this end, Arranz-Romera et al. designed PLGA-based MSs for co-delivery in the treatment of glaucoma. Three molecules with anti-inflammatory and neuroprotective activity were incorporated into the microsystems: MEL, DX, and coenzyme Q10. Neuroprotective activity was tested in vitro on a model of glutamate-mediated neurotoxicity in a retinal cell line and in vivo on a Morrinson rodent model of ocular hypertension. Multi-loaded MSs ensured the simultaneous and controlled release of the three active molecules, reducing the amount of carrier to be injected compared to formulations loaded with single drugs [34]. This multi-therapy strategy has proven useful in treating a multifactorial disease such as glaucoma, and its use has also been investigated in another model of retinal degeneration. In this case, MSs of PLGA were used for the co-delivery of glial cell line-derived neurotrophic factor (GDNF) alone or in combination with MEL. To improve encapsulation and to modulate the release of the biotechnological agent, vitamin E was incorporated into the polymer matrix. The results of the study showed that a single intravitreal injection of MS 0.5 mg in rhodopsin (rho(−/−) knockout mice was able to promote the anatomical and functional rescue of photoreceptors. In addition, it was reported that the combination of MEL with GDNF reduced the initial burst and the rate of delivery and prolonged the release of the protein factor [39].

The neuroprotective efficacy of MEL administered intravitreally in a model of retinal degeneration was also tested by Del Valle Bessone et al [38]. In vitro and in vivo tests showed that the saline solution of MEL, in addition to exerting the neuroprotective effect by increasing the survival of RGCs subjected to oxidative stress, was able to significantly reduce apoptotic processes [38]. Given the promising results of this work, the following year the same authors focused their attention on improving the delivery system. Thus, the research was focused on the retinal delivery of MEL using nanometric systems for noninvasive topical delivery. To promote transcorneal permeation, ethyl cellulose was selected for its mucoadhesive properties as the main constituent of NPs. Numerous benefits have been obtained from encapsulating MEL in NPs, from a slower release profile to increased drug permeation and improved absorption into intraocular structures, providing superior performance in terms of neuroprotection in the retinal degeneration model [40].

Given the therapeutic value the molecule has shown in the DME model, the use of MEL was also studied in a diabetic retinopathy model. To improve the bioavailability of a topical formulation for MEL delivery, next-generation hybrid NPs have been proposed. With the aim of providing mucopenetrating and mucoadhesive properties to the nanosystems, the PLGA-PEG copolymer and two cationic lipids (cetyltrimethylammonium bromide (CTAB) and DDAB) were employed. In order to optimize the encapsulation efficiency and zeta potential of the platforms, different lipid and MEL concentrations were examined according to an experimental design. Lipid coating provided mucoadhesive properties to the systems and a controlled and sustained release of MEL for up to 8 days. In vitro studies showed that MEL loading in hybrid NPs enhanced the neuroprotective and antioxidant efficacy of the molecule. Meanwhile, in vivo tests confirmed that topical instillation did not result in any signs of ocular irritation [41].

The use of the cationic lipids CTAB and DDAB to functionalize systems for MEL ophthalmic delivery was also explored by Carbone et al. Here, polylactic acid (PLA) nanocapsules were prepared with and without lipid coating using a solvent-free method, phase inversion temperature. The study showed that the lipid coating affected four important properties of the nanosystems, referred to as the “4S” (the average particle size, surface charge, shape, and stability) [42].

Recently, MEL has been delivered in protein nanosystems based on human serum albumin (HSA). Although albumin NPs were promising ocular delivery systems, they were susceptible to instability. Therefore, two strategies have been investigated to improve the stability of these carriers, the addition of Eudragit^®^ S100 as a crosslinking agent and thermal stabilization. The latter method proved to be more efficient because, besides being easy to implement and avoiding the use of toxic crosslinking agents, it ensured the physical stability of the NPs at different pH levels [43].

To overcome the limitations associated with the poor stability, solubility, and ocular absorption of MEL, Ahn et al. complexed the molecule with different cyclodextrins (CDs): α, β, γ, and 2-hydroxypropyl-β-CD (HPβCD). Of these, it was reported that HPβCD increased MEL delivery into the rabbit cornea 2-fold, ensuring greater therapeutic efficacy and proving to be a promising therapy for GCD2 [44].

### 3.3. Patent Studies

The use of MEL in treating ocular disorders has garnered increased attention, with its inclusion in patents serving various purposes. Table 2 details patents incorporating MEL. An Australian patent discusses injectable, biodegradable polymeric formulations for the controlled release of active molecules, targeting chronic degenerative diseases like AMD. Active agents in the patent range from monoclonal antibodies to coagulation factors, cytokines, growth factors, peptides, and hormones like MEL. The delivery polymers are triethylene carbonate-based with PLA and/or PGA molar fractions, supplemented with PEG, PVA, and poloxamers to enhance injectability and modulate release. Nanoparticle systems using copolymers improve targeted delivery, combined with injectable hydrogel for simultaneous administration via a double syringe. Release durations vary from 30 days to over 6 months/one year post-ITV injection, showing no anatomical changes and a safe profile.

A U.S. patent describes drug product compositions with implantable elements containing active cells (e.g., engineered RPE cells) or derivatives, capable of producing therapeutic agents for CNS, eye, and spinal disorders. Therapeutic agents include nucleic acids, polypeptides, sugars, small molecules, and MEL. The implantable element can be administered as an implant or injection, with evaluation based on cell viability and therapeutic agent production levels at intervals post-administration. A similar patent was filed at the WIPO in Geneva the year before the publication mentioned. The key difference was in the active cell, specifically mesenchymal stem function cells (MSFCs) or its derivatives. Despite this difference, other aspects such as indications, therapeutic agents, and implementation forms remained identical [53].

A patent filing detailed the use of nanoparticle formulations to deliver oligonucleotides and synthetic RNA for targeted gene expression enhancement. Materials like PLGA, cationic lipids, anionic lipids, or PEG-conjugated lipids were used for fabrication, resulting in nanostructures with diameters ranging from 20 to 150 nm. The lipid-to-oligonucleotide ratios varied from 1:1 to 50:1. The protection of RNA from degradation, particularly by exonucleases, was a key focus. Targeted delivery was achieved through ligands like antibodies, drugs, proteins, and neurotransmitters, with MEL as a specific ligand for receptor targeting. Various administration routes were explored, including ocular, intranasal, oral, pulmonary, and intravenous routes, depending on the desired local or systemic effect. Formulations could take the form of drops, sprays, tablets, capsules, syrups, or solutions. For ocular delivery, topical instillation, subconjunctival application, or intraocular injection were considered. Final formulations could include mucomimetic agents, preservatives, and pH adjusters for stability and efficacy [48].

A patent filed in WIPO details formulations of cannabidiol derivatives targeting cannabinoid type 2 receptors to treat various conditions, including demyelination-associated disorders. These patented formulations are delivered using pharmaceutical vehicles enhancing solubility and bioavailability. The delivery methods include aqueous solvents, co-solvents, cyclodextrin complexes, lipid carriers, or their combinations. Compositions contain stabilizing agents, emulsifiers, polymers, antioxidants like MEL, or a mix. Liposomes and polymeric reservoirs ensure slow and sustained release. These modified-release formulations can be administered topically, intraocularly, orally, or parenterally. Intraocular indications include neuromyelitis optica and Leber’s optic atrophy [49].

A patent deposition in WIPO detailed a novel approach for treating glaucoma using a solid complex combined with γ-cyclodextrin. The document outlined the utilization of biodegradable polymer matrices like PLA and PLGA as carriers for this complex. The degradation and release kinetics of the carrier agent were influenced by the hydrophilic/hydrophobic nature of the end functional groups, leading to controlled release over 1 to 6 months. The implant’s specifications (weight, diameter, length) varied based on its placement in the eye (intracameral or intravitreal). Apart from polymers and therapeutic agents, the formulation could include excipients such as stabilizers, preservatives, antioxidants (like MEL), and buffering or chelating agents, among others [57].

## 4. Discussion

Although the role of MEL in ocular diseases has been widely investigated, the main obstacle of ocular administration is the poor bioavailability in this organ. The eye has several static and dynamic protective barriers that limit the entry and diffusion of therapeutic agents. Ocular barriers do not present the only obstacle to effective administration; other disadvantages that contribute to low bioavailability in ocular tissues are the enzymatic degradation of introduced molecules, poor targeting efficiency, low retention, and permeation time [58]. All these limitations contribute to providing a low dosage to the target tissue. Many ocular diseases are chronic in nature and consequently require sustained amounts of drug. Sustained-release pharmaceutical formulations could maintain constant drug levels at the site of action and partially mitigate this problem [59]. In addition, sustained release could improve patient compliance providing benefits for both topical and intravitreal administration [60]. In the case of topical instillation, it could limit washout, nonproductive systemic absorption, and adverse effects [61]. Concerning intravitreal injection, it could reduce the frequency of administration to ensure therapeutic levels while also limiting the economic costs of hospitalization. In this regard, the use of reviewed delivery systems has been helpful in providing formulations with sustained release and improved bioavailability. The composition of these systems influenced several technological parameters such as the encapsulation efficiency, loading, and drug release pattern [62]. In the case of polymeric delivery devices, polymer selection could be crucial to achieve degradation rates pertinent to the desired release profiles [63]. These vehicles have additional advantages, such as the ability to promote the crossing of precorneal barriers by surface modification with mucoadhesive, viscosifying, and penetration enhancing agents [64,65,66]. Some polymer and lipid matrices themselves exhibit mucoadhesive or mucopenetrating characteristics that enhance trans-scleral absorption and improve the delivery of the active molecule into intraocular tissues [67,68]. In other cases, coating with cationic molecules (CTAB and DDAB) or mucopenetrating polymers (PEG) has been useful for decorating surfaces to enhance electrostatic interaction with the ocular mucosa and penetration of precorneal barriers [69,70,71,72]. In addition, coating surfaces with hydrophilic polymers such as PEG imparted “stealth” properties to delivery vehicles, reducing clearance and increasing therapeutic efficacy [73]. The current systematic review showed that these manufacturing strategies provided several advantages that collectively improved drug delivery to target tissues, bioavailability, and pharmacological activity [74,75].

### Future Perspectives

Eye diseases afflict millions of people worldwide and have an increasing impact on quality of life. Given the increase in visual impairment in the world’s population, a major challenge for researchers is directed toward the development of innovative eye delivery systems to improve the management of these disorders [76].

Although many published and reviewed studies have demonstrated the efficacy of these formulations, it is difficult for them to gain market availability. To date, available clinical studies on the use of MEL for the treatment of ocular disorders are based on oral administration. This issue may lie in the lack of robustness of the preclinical models investigated.

In preclinical testing, it is complicated to select and reproduce disease models; one of the main difficulties consists in the complexity of the pathology. Often, ocular diseases are multifactorial; therefore, several aspects should be captured for consideration. To improve this aspect, the choice of preclinical endpoints should be as close as possible to that of commonly evaluated clinical endpoints [77]. Another aspect that should be considered in translating preclinical results to clinical is the cross-reactivity between species. Laboratory animals used in in vivo studies are small compared with humans, and drug delivery studies in larger animals are often prone to failure because of more complex physiological conditions that are closer to humans. The eyes of the rodent and rabbit, which are the most used animals for this type of preclinical experimentation, have anatomy and physiology that are not representative and predictive of the human eye [78,79,80].

Considering the principle of 3Rs (reduction, replacement, and refinement) before moving to in vivo experimentation on large primates, it might be useful to implement advanced, elegantly designed, and more human-like ex vivo models [81]. Another recently introduced strategy useful for supporting the 3R principle consists of in silico approaches. Using these computational models, it has been possible to generate mathematical computer simulations to accurately reproduce chemical–biological phenomena. The use of “qualified” computational methods has also been recognized by regulatory agencies, which have begun to receive and accept evidence obtained in silico for marketing the approval of new medicinal products [82].

The combination of these shrewdnesses could reduce the deep gap between preclinical and clinical trials to achieve successful formulations in the development of future therapies.

## 5. Conclusions

This review provided a summary of formulations for the ocular delivery of MEL. Several ocular delivery limitations have been overcome using nano- and micrometer-sized delivery systems, including the improved permeation of the molecule into intraocular tissues, and sustained and controlled release profiles over time. The most strategic approaches to improve these constraints involved the fabrication of mucoadhesive and mucopenetrating formulations to cross ocular barriers and increase the absorption and ocular bioavailability of MEL. Although preclinical studies have demonstrated the efficacy of developed formulations, a huge gap has been observed with respect to the number of formulations patented or commercially available. To reduce this gap, it might be useful to revise the preclinical experimental phase by selecting endpoints close to clinical ones and implementing the use of advanced ex vivo models and in silico studies to support the 3R principle while ensuring predictive trials of efficacy in humans.

## Figures and Tables

**Table 1:** Summary of studies on melatonin-loaded ophthalmic formulations.

Therapeutical Indication	Type of Formulation	Route of Administration	Materials	Loaded Drug/Concentration	Size, PDI, ZP, EE%	In Vivo/Animal Model	Effects/Properties	References
Glaucoma	Nanoparticles (NPs)	Topical	PLGA, PLGA-PEG, Boeringher Ingelheim KG (Ingelheim am Rhein, Germany)	1, 3 and 5% (*w*/*w*) to polymer	Size < 300 nm PDI < 0.25 ZP from −8.2 to −32.2 mV EE% from 44.15 to 78.20%	Yes, Male albino rabbits of New Zealand	NPs showed suitable physicochemical features and a sustained in vitro release profile. The tested NPs showed good ocular tolerability in vivo. MEL-PLGA-PEG NPs were the most effective in reducing IOP.	[32]
	Cationic solid lipid nanoparticles (cSLNs)	Topical	Softisan^®^ 100, by Sasol GmbH (Hamburg, Germany) DDAB, SA or PA, Sigma–Aldrich (Milan, Italy)	0.05% (*w*/*v*)	Size = 150–300 nm PDI < 0.3 ZP from −3.99 to +61 mV EE% = 83–96%	Yes, Male albino rabbits of New Zealand	MEL elicited a significant IOP reduction in the rabbit eye. SLN tested in vivo demonstrated good tolerability. SLN with SA was the most effective in terms of IOP reduction.	[33]
	Microspheres (MSs)	Intravitreal (ITV) injection	PLGA, Evonik España (Granollers, Spain)	MEL = 45.80 µg/mg MSs Coenzyme Q10, Sigma-Aldrich (St. Louis, MO., USA) (CoQ10) = 35.71 µg/mg MSs Dexamethasone (DX) = 115.86 µg/mg MSs	Size: 29.04 μm EE%: CoQ10 = 96.42%; DX = 78.20%; MEL = 61.83%	Yes, Adult male Dark Agouti rats	MSs were able to co-deliver the active compounds in a sustained manner over 30 days with low burst release. In vitro studies showed MSs to be neuroprotective in a glutamate-induced cytotoxicity model in the R28 cell line. In vivo efficacy studies were performed using a well-established rodent model of chronic ocular hypertension (OHT), comparing multi-loaded and single-drug-loaded MSs. Multi-loaded MSs showed a significantly neuroprotective effect on retinal ganglion cells.	[34]
	Nanomicelles	Topical	Soluplus^®^, BASF, Ludwigshafen, Germany	MEL and agomelatine, Molekula, Darlington, UK 0.4–0.8% (*w*/*v*)	Size = 61 nm PDI = 0.068	Yes, Rats Sprague Dawley	MEL formulated in nanomicelles showed a longer-lasting hypotonizing effect on IOP than MEL in saline solution. The duration of the hypotonizing effect was further increased with the addition of natural antioxidant lipoic acid.	[35]
	NPs	Topical	PLGA	MEL 5% (*w*/*v*) Dexamethasone, Sigma Aldrich (Shanghai, China) (Dex) 5% (*w*/*v*)	Size = 468 nm PDI = 0.283 ZP = −21.7 mV EE% MEL = 87.25% EE% Dex = 89.41%	Yes, 5-week-old rabbits	NPs showed a core-shell type structure, with Dex encapsulated in the shell and MEL in the core. MEL release from NPs was found to be sustained and were nontoxic in R28 cells. Increased corneal penetration was obtained in comparison to free Dex and ML. Multi-loaded NPs were found to be effective in reducing IOP.	[36]
	Nanomicelles	Topical	Soluplus^®^	MEL and agomelatine 0.4% (*w*/*v*)	×	Yes, Rats Sprague Dawley	Both MEL and agomelatine penetrate the posterior segment of the eye. IOP elevation was drastically reduced by MEL/agomelatine with higher efficacy than that of timolol or brimonidine. Inflammation and apoptosis were partially prevented, thus leading to RGC survival and recovered retinal dysfunction.	[37]
Retinal degeneration	Injectable solution	ITV injection	×	1 mg/mL	×	Yes, New Zealand albino rabbits	An oxidative toxicity model was used to evaluate the neuroprotective effect of MEL on RGCs. According to the results obtained from viability studies on RGCs, the antiapoptotic effect, and the ultrastructural analysis of the retina, MEL proved to be an efficient neuroprotectant.	[38]
	MSs	ITV injection	PLGA, Resomer^®^503, Boehringer Ingelheim Pharma GmbH & Co, Ingelheim, Germany vitamin E, Sigma-Aldrich, Schnelldorf, Germany	MEL, Sigma-Aldrich, Schnelldorf, Germany 10% (*w*/*w*) to polymer Glial cell line-derived neurotrophic factor (GDNF, R&D, Systems, Minneapolis, MN, USA) 0.01% (*w*/*w*)to polymer	Size = 21.6–24.6 µm EE% GDNF = 26.4–39.5% EE% MEL = 39.2%	Yes, Rhodopsin null mice	MSs were able to co-deliver therapeutic active substances in a sustained manner. ITV injection led to the partial functional and structural rescue of photoreceptors in rhodopsin knockout mice. No significant intraocular inflammatory reaction was observed.	[39]
	NPs	Topical	Ethylcellulose, Colorcon^®^ (Buenos Aires, Argentina)	1–2 mg/mL	Size = 147–179 nm; PDI = 0.092–0.2; ZP = −30 mV; EE% = 67–73%	Yes, New Zealand white female rabbits	The in vitro release of MEL loaded in NPs was slower compared to the MEL solution. The increased penetration of MEL-loaded NPs through the cornea was demonstrated by ex vivo and in vivo tests. Efficient neuroprotective activity in a model of retinal degeneration.	[40]
	NPs	Topical	PLGA-PEG, DDAB, CTAB, Merck Life Science S.r.L. (Milan, Italy)	1% (*w*/*w*) to polymer	Size = 190 nm PDI = 0.2 ZP = 39.8 mV EE% = 79.85%	Yes, Male New Zealand albino rabbits	NPs showed good mucoadhesive properties and a controlled release profile. An in vitro study on a model of diabetic retinopathy demonstrated neuroprotective and antioxidant activities. An in vivo study confirmed no signs of ocular irritation.	[41]
Ophthalmic delivery	Polymeric and hybrid aqueous-core nanocapsules (NCs)	×	PLA, Sigma (Milan, Italy) TCT, Farmalabor (Bari, Italy) DDAB, Sigma (Milan, Italy) CTAB, Sigma (Milan, Italy)	0.03% (*w*/*w*)	Size < 250 nm PDI < 0.2 ZP from −36 to +30 mV EE% = 87–90%	No in vivo studies have been conducted	DDAB is the most suitable coating material for the preparation of aqueous-core hybrid NCs. Cationic lipid allowed for the formation of very stable well-defined NCs with a non-spherical shape with sustained and prolonged drug release.	[42]
	NPs	Topical and subconjunctival	HAS, Laboratorio de Hemoderivados UNC (Cordoba, Argentina)	1 mg/mL	Size = 160–180 nm PDI = 0.06 ZP = from −30 to −40 mV EE% = 18–21%	Yes, New Zealand rabbits	The NPs showed a nanometric size, spherical shape, sustained drug release, and physical stability. The topical administration of the NPs produced no irritation, while subconjunctival administration resulted in mild irritation 24 h after administration.	[43]
Granular corneal dystrophy type 2 (GCD2)	Eye drop	Topical	HPβCD, Alfa Aesar (Haverhill, MA, USA)	2.75 mg/mL	×	Yes, New Zealand albino rabbits	Stability studies for 60 days showed no significant change in the pH, osmolarity, and MEL content. HPβCD can reduce rabbit eye irritation and increase MEL delivery into the rabbit cornea 2-fold.	[44]
Diabetic macular edema (DME)	Solution	×	×	0.1, 0.5, and 1 mM	×	No in vivo studies have been conducted	MEL prevented cell hyperpermeability and external barrier disruption. MEL modulated gene expression to maintain mitochondrial homeostasis. MEL prevented the apoptosis of retinal pigmented epithelial cells. MEL may have therapeutic value in DME.	[45]
Optic neuritis	Pellet	Subcutaneous	Vegetable oils, Sigma Chemical Co. (St Louis, MO, USA)	20 mg	2.5 mm × 1 mm (diameter × length)	Yes, Male Wistar rats	Experimental optic neuritis caused alterations in circadian physiology, and melatonin restored the circadian system misalignment.	[46]
Dry eye disease (DED)	NPs	Topical	Polydopamine	MEL 20% tavilermide (Tav) 2%	Size < 250 nm ZP from −20 to +5	Yes, Female BALB/c mice	NPs showed good cytocompatibility and decreased the ROS level and apoptosis ratio. Tav promoted the production of mucins on the ocular surface increasing the retention time of NPs. NPs could effectively treat DED.	[47]

Poly(D,L-lactide-co-glycolide) (PLGA); PLGA-poly(ethylenglycole) (PLGA-PEG); stearic (SA); palmitic acid (PA); cetyltrimethylammonium bromide (CTAB); didodecyldimethylammonium bromide (DDAB); polylactic acid (PLA); Caprylic/Capric Triglyceride (TCT); human serum albumin (HSA); 2-hydroxypropyl-β-cyclodextrins complex (HPβCD). × = no information or not relevant information.

**Table 2:** Summary of patent studies on melatonin-loaded ophthalmic formulations.

Background	Targeted Diseases	Using MEL	Applicant/Manufacturer/Assignee	Patent Number	Publication Date	Reference
Biodegradable injectable polymer formulations that permit an extended or sustained release of a bioactive agent.	Chronic ocular disorder such as age-related macular degeneration (AMD) and others.	Bioactive agents include, but are not limited to, bevacizumab, ranibizumab, aflibercept, and lampalizumab. Additional bioactive agents are hormones like MEL.	W. L. Gore & Associates, Inc., Newark, DE, USA	AU 2,021,277,631 A1	23 December 2021	[53]
Methods, compositions, and implantable elements comprising active cells (e.g., an engineered RPE cell) which may express a therapeutic agent useful for the treatment of a disease.	Neurodegenerative disorders and others.	The therapeutic agent may be any biological substance, such as nucleic acid, a polypeptide (like MEL), a lipid, a sugar, or a small molecule.	SIGILON THERAPEUTICS, Inc., Cambridge, MA, USA	US 20,200,263,196 A1	20 August 2020	[54]
Methods, compositions, and implantable elements comprising stem cells (e.g., a mesenchymal stem function cell (MSFC)) which produce a therapeutic agent.	Neurodegenerative disorders and others.	The therapeutic agent may be any biological substance, such as a nucleic acid, a polypeptide (like MEL), a lipid, a sugar, or a small molecule.	SIGILON THERAPEUTICS, Inc., Cambridge, MA, USA	WO 2,019,195,056 A1	10 October 2019	[55]
Nanoparticle formulations for nucleic acid complexed delivery for increasing gene expression in a targeted manner.	Diseases treatable with oligonucleotides targeting associated mRNA (intraocular melanoma, ocular cicatricial pemphigoid, optic neuritis, neuromyelitis optica).	Ligands (such as MEL or others) can be conjugated to the particle to target the nucleic acid complex to a specific cell type or tissue of interest.	Translate Bio MA, Inc., Lexington, MA, USA	US 20,180,311,176 A1	1 November 2018	[48]
The topical delivery of therapeutic agents using cell-penetrating peptides.	AMD and other eye diseases.	The therapeutic agent is or includes an anti-dyslipidemic agent, an antioxidant (such as MEL), an anti-inflammatory agent, a complement inhibitor, a neuroprotector or an anti-angiogenic agent, or any combination thereof.	MacRegen, Inc., Birmingham, AL, USA	US 20,210,085,797 A1	25 March 2021	[56]
Formulations of cannabidiol derivatives and their use as modulators of cannabinoid receptor type 2 in the treatment of various diseases.	Demyelinating diseases (such as neuromyelitis optica) and others.	Pharmaceutical vehicle optionally comprises a stabilizer, emulsifier, polymer, antioxidant (such as MEL), or any combination thereof.	EMERALD HEALTH PHARMACEUTICALS Inc., San Diego, CA, USA	WO 2,020,163,612 A1	13 August 2020	[49]
Implants of solid complexes for the treatment of ophthalmic diseases.	Glaucoma and others.	To improve the stability of the therapeutic agent, the intraocular implant may comprise excipients like preservatives, an antioxidant (such as MEL), buffering agents, chelating agents, electrolytes, and others.	ALLERGAN, Inc., Irvine, CA, USA	WO 2,019,023,211 A1	31 January 2019	[57]

## Data Availability

Not applicable.
